# Distributed Optical Fiber Sensors with Ultrafast Laser Enhanced Rayleigh Backscattering Profiles for Real-Time Monitoring of Solid Oxide Fuel Cell Operations

**DOI:** 10.1038/s41598-017-09934-3

**Published:** 2017-08-24

**Authors:** Aidong Yan, Sheng Huang, Shuo Li, Rongzhang Chen, Paul Ohodnicki, Michael Buric, Shiwoo Lee, Ming-Jun Li, Kevin P. Chen

**Affiliations:** 1 Department of Electrical and Computer Engineering, University of Pittsburgh, Pittsburgh, PA 15260 USA; 2 National Energy Technology Laboratory, 626 Cochrans Mill Road, Pittsburgh, PA 15236 USA; 3 Corning Incorporate, Corning Incorporated, One Riverfront Plaza, Corning, NY 14831 USA

## Abstract

This paper reports a technique to enhance the magnitude and high-temperature stability of Rayleigh back-scattering signals in silica fibers for distributed sensing applications. With femtosecond laser radiation, more than 40-dB enhancement of Rayleigh backscattering signal was generated in silica fibers using 300-nJ laser pulses at 250 kHz repetition rate. The laser-induced Rayleigh scattering defects were found to be stable from the room temperature to 800 °C in hydrogen gas. The Rayleigh scatter at high temperatures was correlated to the formation and modification of nanogratings in the fiber core. Using optical fibers with enhanced Rayleigh backscattering profiles as distributed temperature sensors, we demonstrated *real-time* monitoring of solid oxide fuel cell (SOFC) operations with 5-mm spatial resolution at 800 °C. Information gathered by these fiber sensor tools can be used to verify simulation results or operated in a process-control system to improve the operational efficiency and longevity of SOFC-based energy generation systems.

## Introduction

Solid oxide fuel cell (SOFC) technology is a promising and versatile energy generation scheme. SOFCs are used in a wide variety of applications ranging from clean automobiles to distributed electric power generation systems. SOFCs in stand-alone or hybrid generation configurations can utilize a wide array of gaseous fuels from hydrogen to biogas to achieve high energy conversation efficiencies and low emissions. Modern SOFC system exhibit high internal reaction temperatures, and are capable of internal gas-reforming to provide fuel flexibility and increased versatility. However, the long-term stability of SOFC-systems is primarily hindered by material and structural degradation; which impacts the profitability of large-scale SOFC system deployment. In order to improve the longevity of SOFC systems, we must be able to measure and engineer the temperature distribution inside fuel cell assembly. Temperature variations in an SOFC assembly are a result of convection as gasses with varying thermal conductivities flow through the cell, conduction of heat through the supporting structure, and the heat generated by the distributed internal reforming or oxidation reactions producing electrical output. Each of these processes contributes to temperature variations inside a fuel cell. The temperature variations are responsible for the thermal stresses, which are one of the main issues that impact the lifetimes of SOFCs^1–3^. In addition, the spatial temperature variations also affect the current density distributions in SOFCs through the generation of local over-potentials^4^. Thus, the improvement and optimization of fuel cell lifetime and performance relies on a detailed understanding of temperature profiles inside an SOFC. Currently, numerical simulations are used to estimate temperature distributions in SOFC systems because experimental measurements during operation have been extremely challenging. Thermocouple devices have been used by some researchers to perform single-point measurements^3, 5^. As many as 36 thermocouples have been inserted in a fuel cell stack to perform multi-point measurements^5^. However, each thermocouple requires 2 electrical wires for each point-measurement; each of which introduces additional heat-losses which skews the accuracy of measurements. It is also physically impossible to place thermocouples in extremely close proximity to one another in order to produce high spatial-resolution measurements. Large numbers of wires in the cell could also impede fuel gas-flow, which may further alter the temperature profile during measurement.

Distributed fiber optic sensing is a potentially powerful technique to measure the spatial temperature profile of an operating SOFC system. Being well-suited for harsh environment sensing applications, fiber optic sensors have been widely used for high-temperature measurements. Distributed sensing schemes such as Rayleigh-scattering Optical Frequency Domain Reflectometry (OFDR) can perform distributed temperature sensing using unmodified single-mode optical fiber to achieve <1-cm spatial resolution. One of the key challenges of distributed sensing using the Rayleigh backscattering is the weak Rayleigh-backscattering intensity exhibited by conventional optical fibers. The weak Rayleigh backscattering is hardly a surprise, given that telecommunications optical fibers are designed for low-losses, including low Rayleigh-scattering losses. To address this challenge, several approaches have been attempted to increase Rayleigh scattering in fiber, such as UV exposure of the hydrogen-loaded single mode fiber^6^, isotopes irradiation of Ge/P co-doped fiber^7^, larger scattering cross-sections using polymer fiber^8^. The enhanced Rayleigh scattering profiles result in larger scattering signals at the detector(s) and better spectral correlation quality between the measured high-temperature Rayleigh profile and the reference room-temperature Rayleigh profile. This in turn improves the fidelity of the distributed measurement as well as the useful range of temperatures over which the sensor-fiber can operate effectively. However, even though Rayleigh scattering enhancement may be used to effectively extend the operational temperatures and longevity of fiber sensors, eventually environmental effects will overcome the stability of the measurement during extended periods at high temperatures. Buric *et al*. reported rapid degradation of spectral shift quality of Rayleigh distributed temperature measurements at high temperature up to 800 °C^9^. These changes will eventually compromise sensitivity and reliability of the distributed measurements, although the use of the types of scattering enhancement modifications described here may delay or mitigate the effects of such degradation, yielding a new regime of measurement capability.

In this paper, we propose to use femtosecond ultrafast laser irradiation to produce enhanced Rayleigh scattering profiles in optical fibers that are stable at high temperatures. The ultrafast laser is known to be a useful tool to produce fiber Bragg grating (FBG) point-sensors with superior temperature stability compared to FBGs produced by UV lasers^10^. It’s also used to produce random gratings to increase the light scattering in single-mode fiber for multi-parameter measurements^11^ and the index change result from the formation of self-ordered nanogratings were proved to be stable at high temperature^12-15^. The ultrafast laser fabrication of Bragg gratings requires precise optical alignments for point-by-point grating writing. Alternatively, holographic or interferometric apparatus are needed to form periodic refractive index patterns in fiber cores. In contrast, this paper presents a manufacturing method to produce distributed fiber sensors with high spatial resolution using ultrafast laser pulses to perform continuously scan of the fiber cores. It does not require point-by-point periodic laser writing or holographic laser exposure to form fiber Bragg gratings with nanometer precision in grating periods. The resultant fibers with laser-enhanced Rayleigh scattering profiles, due to the formation of nanogratings, can perform distributed temperature sensing with 5-mm spatial resolution at 800 °C in highly reactive fuel gas (hydrogen) stream. Using this powerful sensing tool, we demonstrate distributed temperature measurement in an operating SOFC system. Information gathered by this fiber sensor tool can now be compared with simulation results to aid in SOFC system design, and ultimately improve the operational efficiency and longevity of the SOFC system.

## Results and Discussion

**Figure 1:**
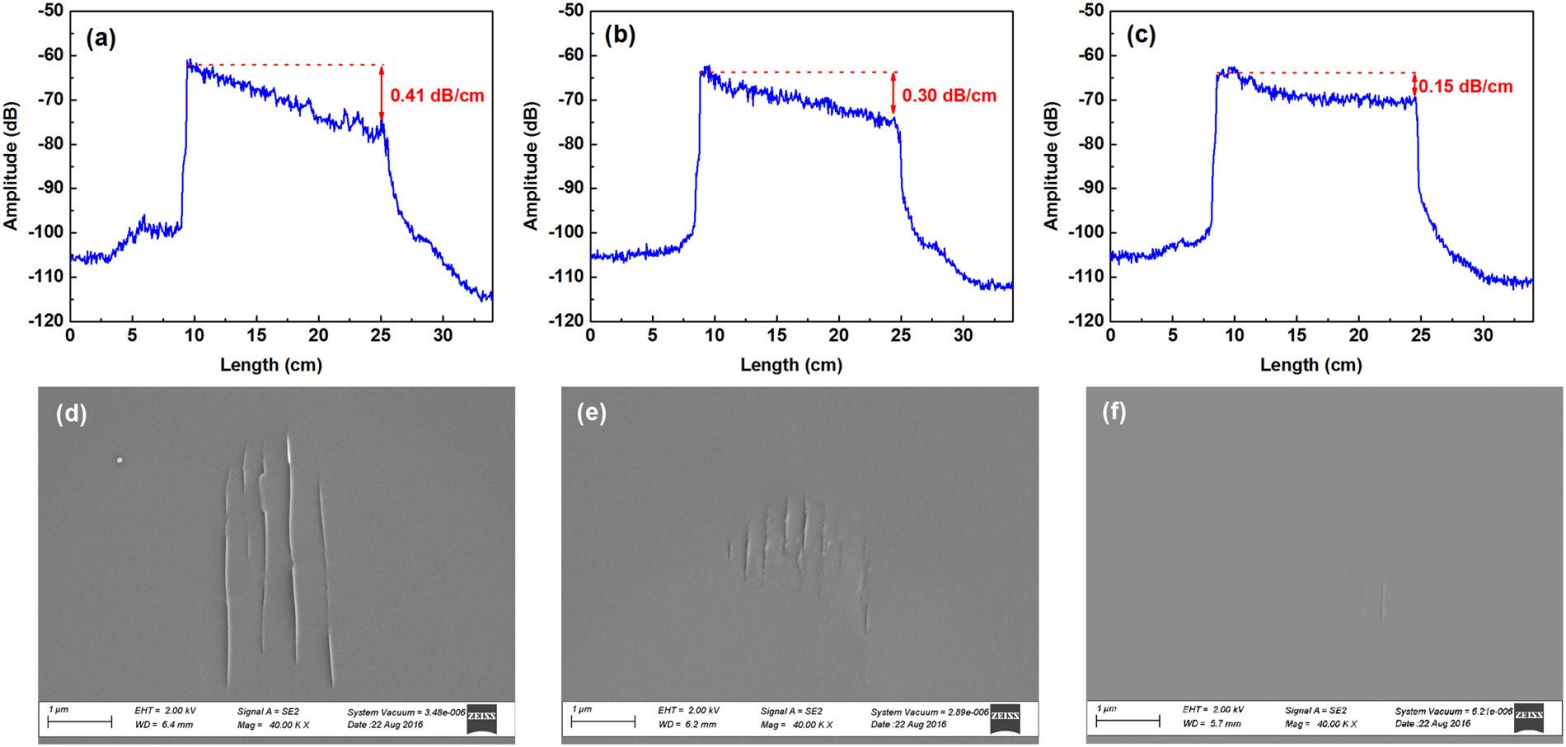
(**a**)–(**c**) Ultrafast laser-enhanced Rayleigh backscattering profiles and (**d**)–(**f**) scanning electron microscope (SEM) images of the cross-sectional morphologies of nanogratings formed at scanning speed of 0.1 mm/s, 0.5 mm/s and 1 mm/s, respectively.

The enhanced Rayleigh backscattering profiles produced in 17-cm long sections of fiber using the ultrafast laser direct writing scheme are shown in Fig. 1. A commercial OFDR interrogator (LUNA OBR4600) was used to measure the Rayleigh backscattering. The fiber translation speeds were 0.1 mm/s (Fig. 1a), 0.5 mm/s (Fig. 1b), and 1 mm/s (Fig. 1c), respectively. An increase in the Rayleigh backscattering amplitude of 40–45 dB was obtained with laser irradiation for all of the chosen writing speeds. The ultrafast laser irradiation did not yield Rayleigh scattering enhancements when the laser writing speed exceeded 2 mm/s. The laser irradiation also introduced significant optical propagation losses in the irradiated fiber-samples; which is characterized by the slope of the Rayleigh-enhanced region. At 300-nJ pulse energy, the average propagation losses in the irradiation sections are 0.41 dB/cm, 0.30 dB/cm and 0.15 dB/cm, respectively. Therefore, a scanning speed of 1 mm/s was the optimal processing condition that minimized the insertion loss of the irradiated sensor-segment.

After testing, the irradiated fiber segments were cleaved and imaged using a Scanning Electron Microscope (SEM - Zeiss Sigma 500 VP). This observation reveals the telltale signs of the mechanism behind the laser-induced Rayleigh enhancements. Figure 1d–f show the SEM cross-section images of the fiber core regions after the laser irradiation was performed at scanning speeds of 0.1 mm/s, 0.5 mm/s, and 1 mm/s, respectively. It is clear that the ultrafast laser enhancement of the Rayleigh profile is closely linked to the formation of laser-induced nano-gratings inside the fiber core. These nanogratings were only observed in fibers which exhibited enhanced Rayleigh back-scattering after processing. At present, several hypotheses have been proposed for the formation mechanisms of nanogratings including interference between the incident light field and the electric field of the induced plasma wave^16^; formation of spherically-shaped nanoplasmas from inhomogeneous hot-spots and their further evolution into periodic nanoplanes^17^; coupling between attractive interaction and self-trapping of exciton-polaritons^18^; and the development of periodically distributed defects near the edges of the irradiated section induced by plasma standing waves^19, 20^. Previous researchers have reported, the volume of laser-induced nanogratings in silica depends on the number of laser pulses and the total pulse energy deposited into the focal volume^20–23^. These induced grating structures result in birefringence because the induced structures are oriented with respect to the induced standing plasma wave. This structural orientation will likely produce polarization-dependent Rayleigh scattering enhancement.

This is also confirmed in Fig. 1d–f with slower fiber translation speed leading to larger nanograting volumes at the same pulse-repetition rate and energy. Periodicities of the nanogratings are measured to be around 500 nm (Fig. 1d) or 250 nm (Fig. 1e,f), which is consistent with wavelength (500-nm) or half wavelength (250-nm) of the ultrafast laser in silica^16, 17, 24, 25^. The height and width of the nanogratings depends on the laser energy deposited into the focal volume, which is also determined by the laser scanning speed. The width of the nanograting plane is measured to be less than 10 nm, and the length is approximately 4 μm at 0.1 mm/s fiber translation speed. The length of the grating is reduced to less than 1 μm with the scanning speed of 1 mm/s. It appears that the volume of the induced nanograting is directly correlated with the increase in propagation loss shown in Fig. 1.

**Figure 2:**
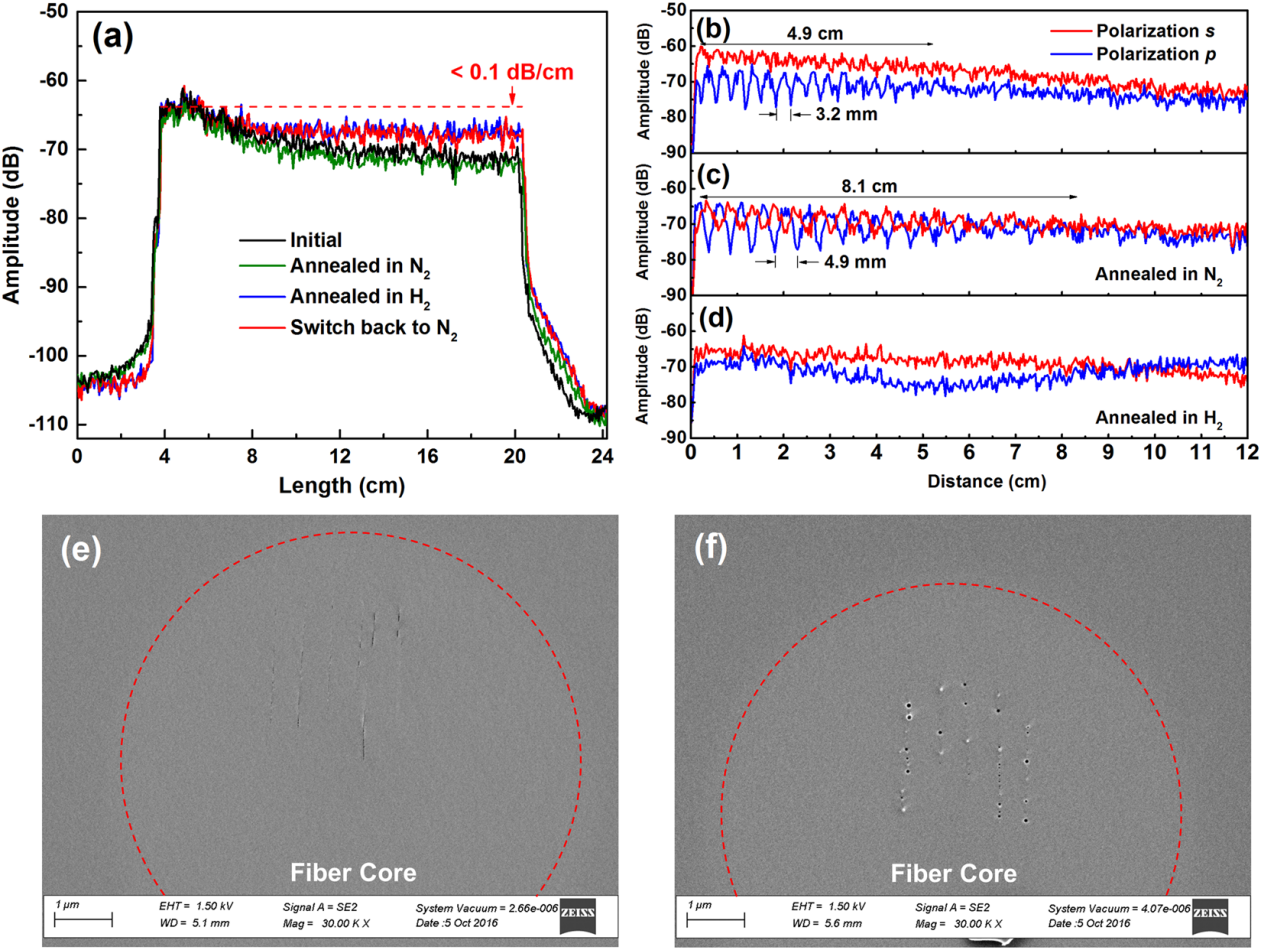
Anneal process at 800 °C in N_2_ and 10% H_2_. (**a**) Rayleigh scattering amplitude measured during annealing in N_2_, and 10% H_2_ (balanced with 90% N_2_), (**b**)–(**d**) scattering signal amplitude of the s and p polarization states emitted by the irradiated fiber before and after high temperature annealing in N_2_ and 10% H_2_ respectively, (**e**)–(**f**) SEM images of nanogratings morphology changes occurring after annealing in N_2_ and 10% H_2_, respectively.

To determine the thermal stability of the laser-enhanced Rayleigh scattering profiles at high temperatures in highly reactive fuel gas environments, the laser irradiated fibers were first annealed in a tube furnace at 800 °C. The tube furnace is flushed with H_2_ balanced with N_2_. Two calibrated mass flow controllers were used to control the concentrations of H_2_ up to 10% in the gas mixture. During the annealing process, the temperature of the furnace was first ramped up from room temperature to 800 °C in air and held at temperature for 4 hours before switching to 10% H_2_. The Rayleigh backscattering profile was monitored to determine the fiber sensor resilience while 10% H_2_ was applied for 4 hours before switching back to air. The scattering profiles measured during the testing are presented in Fig. 2a. The laser-enhanced section exhibits non-uniform Rayleigh intensity profiles where the beginning of the enhanced section have higher scattering amplitude than the end of the section. This is likely a direct result of optical loss from the propagation of the fundamental fiber mode through the laser-induced nanogratings in the fiber core. As the fundamental core mode propagates through the damaged core section, its intensity profile decreases due to the additional scattering loss. This is evident as shown in Fig. 1a,b, while the laser enhanced Rayleigh profiles exhibit a linear slope of 0.41 dB/cm and 0.31 dB/cm, respectively. This loss is closely connected to the formation of nanogratings as shown in Fig. 1d,e. Via careful control of laser writing speed, it is possible to reduce the propagation loss in the enhanced section of the fiber, which results in a relatively flat Rayleigh profile (e.g. Figure 1c). The initial annealing process in N_2_ at 800 °C yielded only a small decrease in the Rayleigh scattering profile. The small initial scattering decrease stabilizes in the first 10 minutes of annealing. Given that the enhancements of Rayleigh scattering are closely tied to the formations of nanograting as shown in Fig. 1, the high temperatures stability of the Rayleigh profile shown in Fig. 2a are consistent with previous observations of temperature resilience of ultrafast laser induced nanogratings^13–15^. The subsequent exposure to 10% H_2_ produced variation in the Rayleigh profile. The scattering profile after the first 4-cm section increase after the 10% H_2_ annealing. This change is permanent in both 10% H_2_ and N_2_ atmosphere as shown in Fig. 2a. At the same time, the average loss of the irradiated fiber decrease to less than 0.1 dB/cm from 0.15 dB/cm. The scattering increase in hydrogen takes place when temperature is higher than 700 °C. This process occurs rapidly and the profile stabilized within 10 minutes in 10% H_2_ atmosphere. When switching back to nitrogen or air after annealing in H_2_, no significant change in scattering was observed as shown in Fig. 2a; indicating that the increase of scattering due to hydrogen at high temperatures is permanent.

In this work, the birefringence of the irradiated fibers was measured and correlated to the thermal annealing process using the OFDR instrument in order to shed light on stability of the laser-induced Rayleigh scattering and its degradation mechanisms. Given that nanogratings formed in the fiber cores are structurally asymmetric, they induce birefringence in negative uniaxial structures^16, 26^. The slow and fast optical axes of the induced gratings were aligned parallel and perpendicular to the nanoplanes, respectively^27^. Figure 2b–d shows the Rayleigh backscattering signal demodulated into *s-* and *p-* polarizations by the OFBRs internal polarizing beam splitter^28^. The irradiated fiber produces scattering along both the fast and the slow axes of the fiber, which is not necessarily aligned with polarization states demodulated by the polarizing beam splitter. This results in birefringence beating as shown in Fig. 2b–d. The fading of the beating fringes over the length of the irradiated section is caused by diminishing coherence of the two polarization modes as the spatial difference between the two modes exceeds the resolution limit of the instrument^28^. The effective beating length of 3.2 mm was measured for the irradiated fiber at the laser scanning speed of 1 mm/s as shown in Fig. 2b at 1.5-μm wavelength. The magnitude of the effective index difference (i.e. modal birefringence *Δn*) can be quantified by $\Delta n=\lambda /{L}_{b}=4.8\times {10}^{-4}$, which agrees with birefringence in type II-IR gratings fabricated by ultrafast lasers in silica^29^. After annealing at 800 °C in N_2_ for 4 hours, the beating length increased and settled to 4.9 mm. This corresponds to a slight reduction of the modal birefringence to 3.2 × 10^−4^ as shown in Fig. 2c. In sharp contrast, exposure in 10% H_2_ at 800 °C quickly reduced the birefringence to < 10^−6^ as shown in Fig. 2d. To determine the origin of this decay in birefringence, SEM photos of the nanogratings in samples annealed in air and H_2_ are reported in Fig. 2e,f. As shown in Fig. 2e, parts of the nanoplane have morphed into several discontinuous shorter segments after annealing in air, which results in a slight decrease in the birefringence. However, for the fiber samples annealed with hydrogen, the nanograting planes evolve into a series of nanopores with diameters around 50-nm (Fig. 2f), this is probably the underlining cause of the decrease of optical loss of the irradiation fiber as shown in Fig. 2a. A possible reason for this pore formation is that the nanograting features in silica are more likely to be densified during exposure to hydrogen at high temperatures^30^. As a result, birefringence for the nanogratings was almost erased after hydrogen exposure.

**Figure 3:**
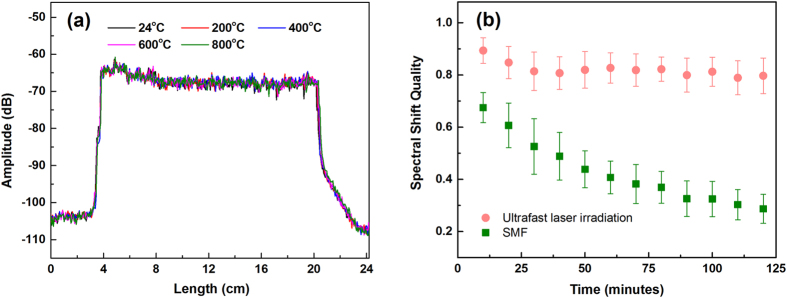
(**a**) Thermal stability of scattering features after an annealing process in 10% hydrogen at 800 °C for 10 minutes and (**b**) comparison of the spectral shift quality vs time at 800 °C for fiber irradiated by the ultrafast laser and un-irradiated standard fiber.

To further characterize the induced Rayleigh scattering stability, the annealed fibers were used as temperature sensors over a wide range of applied temperatures. Temperature changes are measured by the OFDR system by cross-correlating the Rayleigh backscattering profile at a known temperature with the Rayleigh backscattering profile after heating. This cross correlation yields a spectral shift value plotted over the length of the test-section which may be further fitted to the temperature using a calibration curve intrinsic to the fiber material. After the initial thermal annealing in 10% hydrogen at 800 °C for 10 minutes, the Rayleigh scattering enhancement becomes significantly more stable. The scattering profiles measured using the annealed fibers from 24 °C to 800 °C in a 10% H_2_ atmosphere are shown in Fig. 3a. No significant scattering amplitude changes were observed as compared to the post-annealed fiber. Figure 3b shows the comparison of the spectral shift quality between the laser-irradiated fiber and a normal single-mode fiber (Corning SMF-28) at 800 °C. The spectral shift quality is a measure of the strength of correlation between the backscattering profiles in measurement and reference spectra^31^. The parameter is defined as the maximum value of cross-correlation between the measurement and reference spectra normalized by the maximum of the reference spectrum autocorrelation. The reference spectrum was recorded at 800 °C. A spectral shift quality of 1.0 indicates that there is a perfect match between reference and measurement spectra. The shift quality will decrease when the scattering signal becomes significantly different from the reference signal. Results shows that the spectral shift quality at T = 800 °C under 10% hydrogen decreased from 1 to less than 0.3 within 2 hours for Corning SMF-28 fiber, while the spectral shift quality in the fiber with the enhanced scattering profile remained above 0.8 for the same time period. When the irradiated fiber was tested more than 20 hours under the same conditions, the value of the spectral shift quality remained above 0.75. This result shows that distributed temperature measurement can be performed with high fidelity at high temperature over a long period of time; even in a highly reactive hydrogen containing environment.

**Figure 4:**
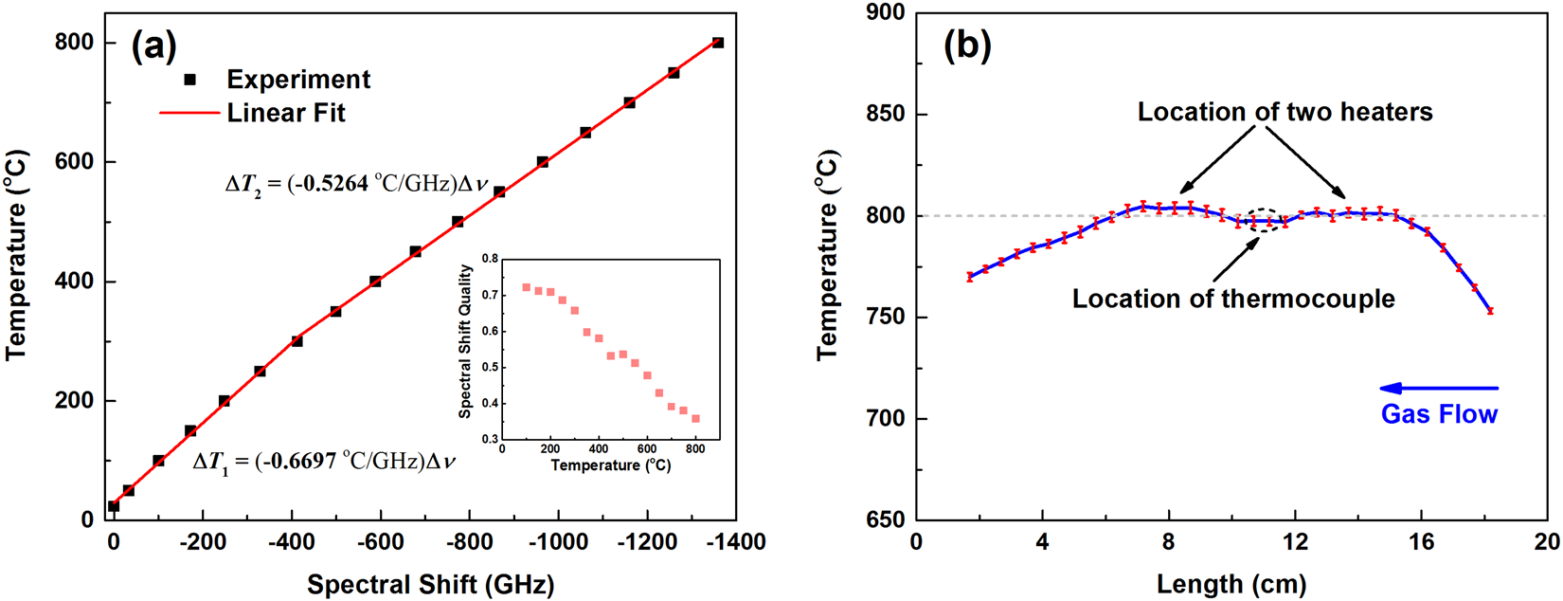
(**a**) Rayleigh spectral shift measured by OFDR at different applied temperatures for fibers with enhanced Rayleigh scattering profile. The inset shows the spectral shift quality vs. temperature. (**b**) Temperature profile of a furnace measured by the distributed fiber sensors with enhanced Rayleigh scattering profile. The furnace temperature was set at 800 °C. The error bar was obtained by 8 different measurements for each with a cooling cycle to room temperature.

The temperature dependence of the spectral shift in the irradiated fiber was characterized over the temperature range from 24 °C to 800 °C. The spectral shift versus temperature change for the ultrafast laser-irradiated fiber is shown in Fig. 4a. There appears to be a change in slope at approximately *T* = 300 °C. Two linear temperature/spectral shift coefficients are obtained in those two respective temperature regimes. Between 23 °C and 300 °C, the temperature change linearly depends on the spectral shift with a slope of −*∂T/∂ν* ~ 0.6697 °C/GHz. While above 300 °C, the coefficient changes to ~ 0.5264 °C/GHz. This thermal-optic coefficient change is caused by the phase transition change in silica around 300 °C^32^. The formation of nanogratings also results in a significant increase in the spectral-shift quality for distributed temperature measurement over a wide temperature range. This is shown in the inset of Fig. 4a. When the Rayleigh profile at 23 °C was taken as the reference, and the temperature was then increased to 800 °C; the spectral shift quality remained above 0.3, which is significantly higher than the threshold spectral-shift quality required for reliable temperature measurement (0.15).

To study the reliability of temperature measurements over a wide range, the Rayleigh enhanced irradiated fiber was tested using a tube furnace. Figure 4b shows the temperature measurement repeatability using Rayleigh-enhanced fiber. The furnace temperature was monitored and controlled by a single thermocouple placed in the center section of the furnace as denoted in the figure. The furnace was heated up by two heaters, which were placed 3.5-cm from the thermocouple location on either side of the center of the furnace. The spatially resolved temperature profile of the furnace at the set temperature of 800 °C is shown in Fig. 4b with a spatial resolution of 0.5 cm. The reference spectrum was taken at 23 °C. Then the temperature of the test furnace was raised to 800 °C (calibrated by the thermocouple sensor installed in the furnace). The temperature profile of the furnace was measured by the distributed fiber sensor and presented in Fig. 4b. To gauge the repeatability of the distributed fiber sensor, the furnace was cooled to the room temperature and repeat the above thermal cycles 8 times. Error bars presented in Fig. 4b, which was calculated by these 8 separated measurements, show a standard deviation less than 4 °C, which highlights the measurement reliability and repeatability. The measurement also reveals that the heater locations have slightly higher temperatures than the location of thermocouple which was regulating the temperature. In the experiment, 20 sccm of 10% hydrogen flows from one side of the furnace’s quartz tube to the other. As a result of the high thermal conductivity of hydrogen gas, larger temperature gradients were observed from the gas injection side (right side in the figure). These fine details of the temperature profile revealed by the distributed fiber sensors highlight the excellent temperature measurement capability which is useful for both the design and control of energy systems.

The thermally-stable distributed fiber-optic sensors demonstrated in this paper provide a powerful tool to perform *real-time* measurements and to understand the operation of high-temperature SOFCs. The fuel cell tests described here were performed at the U.S. Department of Energy’s National Energy Technology Laboratory fuel-cell testing facility (Morgantown, WV). This test-facility was designed for assessing the performance of kw-level SOFC distributed power generation systems. A single commercial planar fuel cell (ASC5, Fuel Cell Materials, Lewis Center, OH) was used for the test. This type of anode-supported fuel cell has a yttria-stabilized zirconia (YSZ) electrolyte, a Ni-YSZ anode, and a La_1−x_Sr_x_Co_1−y_Fe_y_O_3−d_ (LSCF) cathode. The anode support is 5 cm × 5 cm, and the cathode active area is 4 cm × 4 cm.

**Figure 5:**
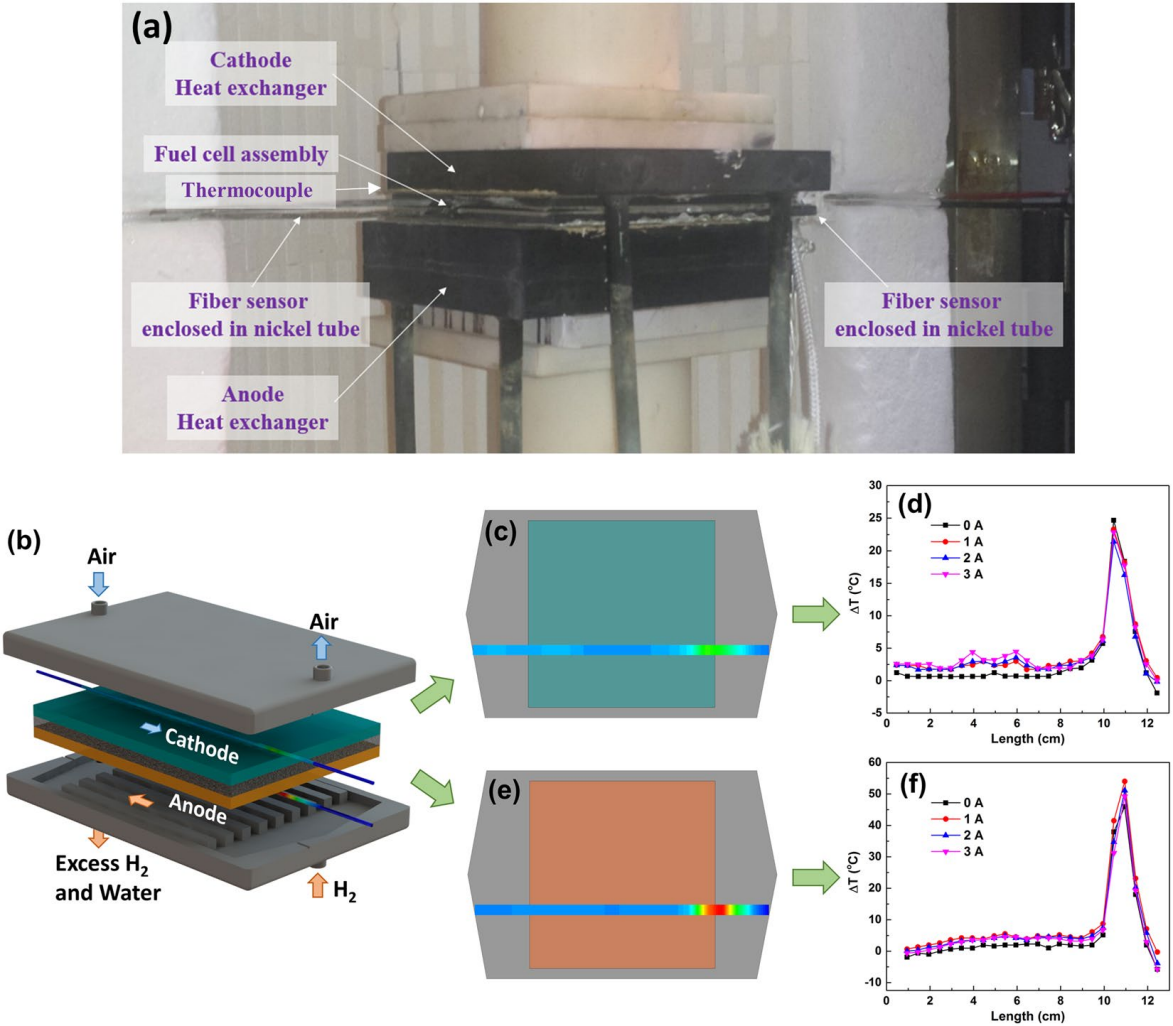
Temperature variation change of the cathode and anode in the solid oxide fuel cell at different current load. (**a**) Photograph of a fuel cell with an inserted fiber sensor, (**b**) schematic of the solid-oxide fuel cell. Distributed temperature measurements in cathode (**b**,**c**) and anode (**e,f**) at current 0 A, 1 A, 2 A, 3 A respectively with 100% hydrogen fuel.

Figure 5a,b shows the photograph and schematic of the SOFC in a counter-flow configuration. Hydrogen fuel flows from the inlet to the outlet across a series of parallel fuel channels, while air flows in parallel channels in the opposite direction on the other side of the cell. The distance from the inlet to outlet is 8 cm and the length of the electrolyte region is 5 cm. In order to probe the temperature change at different electric loads, fiber sensors were placed in the anode channel and cathode channel respectively, as shown in Fig. 5a,b. Before installing fiber sensors, the fuel cell assembly was heated up to 860 °C to form a gas-tight seal using glass-ceramic sealant. The completed cell was then aged at 750 °C for 6 h to reduce the anode material under 100% H_2_. After checking the normality of fuel cell operation, the assembly was brought down to room temperature and fiber sensors were inserted into the cell through nickel tubes which provided access to the cell-body and encased the fiber-sensor outside the cell-body. Both ends of the tubes positioned outside of the furnace were sealed with silicone putty where the fiber entered or exited the assembly. Prior to the operation, the sensor-embedded fuel cell was heated externally to ~ 720 °C, which is confirmed by both point thermocouple devices and the distributed fiber sensors. The distributed temperature profile of the cell was continuously monitored and compared with the reference during heating. Once the SOFC reached operational temperature, 500 sccm of 100% hydrogen was injected into the anode and 500 sccm of air was also injected into the cathode. When pure hydrogen was introduced at the anode, the temperature around the fuel gas inlet increased dramatically as shown in Fig. 5c–f with 5-mm gauge length. At the anode side, the temperature around the fuel inlet increase up to 55 °C above ambient. The temperature peaks near the border area of the electrolyte area. This is caused primarily by high thermal conductivity of hydrogen fuel. The electrolyte has significant influence on the temperature profile on the cathode side, where the temperature rise was much lower at about 25 °C. The temperature variation induced under different electric loads was also measured with load-current ranging from 0 A to 3 A. The current loading resulted in a slight temperature increase near the center section of the fuel cell. At a 3-A current, the temperature increase on both anode and cathode sides was about 5 °C. While this reaction-induced temperature profile change might not be a significant issue for a single SOFC operating with external applied heating, the cumulative induced temperature effects on stacked self-heated fuel cell assemblies should be carefully studied. The fuel-gas induced temperature changes inside the fuel cell depend strongly on the flow rate of hydrogen and its concentration. We note anecdotally that a mixture of 4% H_2_ does not exhibit significant convective heating effects on the SOFC assembly, while a 10% mixture induces readily observable convective effects. Given that the convective impacts of fuel gas streams on temperature profiles at the anode and cathode are significantly different, designing and implementing an optimal temperature control scheme will require modelling of the thermal characteristics of the SOFC assembly, along with the thermal and chemical properties of the cell materials and applied gases. The distributed fiber sensor demonstrated in this paper provides a new and valuable tool to measure the constantly-evolving SOFC temperature profile, which will lead us towards optimizing the efficiency and longevity of SOFC-based energy systems in the future.

## Conclusion

While the ultrafast lasers have been used previously to fabricate FBG point sensors, this paper presents a far simpler technique to produce distributed fiber-sensors to perform continuous temperature sensing with 5-mm spatial resolution in high temperature, highly reactive hydrogen-containing environments.

By employing femtosecond laser irradiation with sub -μJ pulses, we demonstrated that the Rayleigh scattering profile from commercially available silica fibers can be enhanced by more than 40-dB through nanograting formations in the fiber core. This increases the available measurable intensity at the optical detectors, leading to significant improvements in both the SNR and spectral shift quality of an OFDR-based measurement. The new Rayleigh backscatter features induced by femtosecond laser irradiation are stable at high temperatures, which enables reliable temperature measurements in extreme environments. The ultrafast laser-induced optical loss (~0.1 dB/cm) might be significant for distributed sensing measurements with long interrogation lengths. However, for a wide array of harsh environment energy applications with interrogation length less than 2-m (20-dB overall loss vs. 40 dB signal enhancements), this technique represents a powerful new tool to potentially study a wide arrange of energy production processes from biomass reactors, to solid oxide fuel cells, to monitoring in nuclear reactors. Using this distributed sensing tool, reliable temperature measurements were achieved from room temperature to 800 °C. The system was used to probe an operating SOFC’s temperature dependence on fuel stream inlet chemistry and fuel utilization that was previously inaccessible. The capability for *in-situ* temperature monitoring with high spatial resolution within operational energy conversion devices such as solid oxide fuel cells represents a significant opportunity for process efficiency and long-term stability; which are two key metrics required for enabling widespread deployment of SOFCs in the power generation sector. We believe the system described here will also be useful for measurements in existing harsh-environment energy systems including combustion systems, boilers, and gas turbines. Distributed high-temperature sensor technology represents a major opportunity for potentially improved efficiencies, mitigating against undesirable emissions, improving safety, and reducing the cost of electricity to the consumer by decreasing maintenance and downtime.

## Method

**Figure 6:**
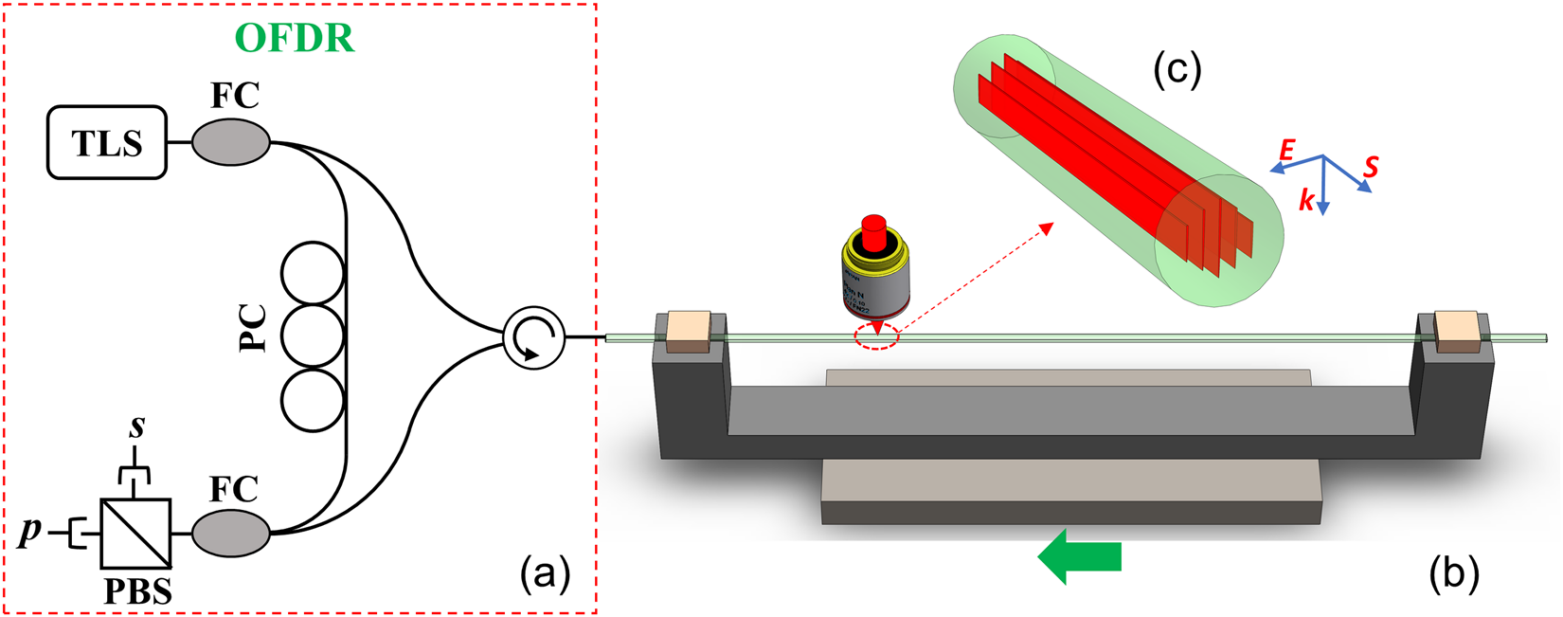
Schematic sketch of the Rayleigh Enhancement setup. (**a**) OFDR system (LUNA OBR 4600 with internal components^33^: TLS: tunable laser source, FC: Fiber Coupler PC: polarization controller, PBS: polarizing beam splitter). (**b**) Schematic sketch of the ultrafast laser irradiation on optical fibers. (**c**) Nanograting formed during laser irradiation with S: The direction of laser scanning, E: The direction of the electrical field. *k*: nanograting orientation and the direction of light propagation.

The schematic diagram of the experimental method is shown in Fig. 6. The ultrafast laser system consists of a Coherent MIRA-D Ti: sapphire seed oscillator and a RegA 9000 regenerative amplifier operate at 800 nm with a repetition rate of 250 kHz. The pulse width was adjusted to 300-fs. The on-target pulse energy was set at 300-nJ, which was determined to be slightly above the threshold pulse energy required to enhance the Rayleigh backscattering of the fiber. A cylindrical telescope was used to shape the laser beam and control the shape of the focal volume. An 80× aberration-corrected microscope objective (NA = 0.75) was used to focus the beam. Oil immersed objectives (80×) were used to process cylindrical shaped fibers. The protection jacket of the fiber was removed with the length of 18 cm before the irradiation process. The laser beam was focused to the center of the fiber core. The beam waist diameter was estimated to be ~2 μm. The fiber being irradiated is also connected to a commercial OFDR interrogator (LUNA OBR4600). The Rayleigh backscattering profiles were monitored by the interrogator during the laser irradiation process. During the laser irradiation, the optical fiber samples were firmly mounted on a computer-controlled air-bearing motion stage (Aerotech ABL2002) and translated in the direction perpendicular to both the irradiation beam and the laser polarization. The laser was operated with a continuous pulse-train while the fiber was translated over 20-cm longitudinally. The translating speed of the stage varied from 0.1 mm/s to 1 mm/s, which allows adjustment of the effective number of pulses contributing to the formation of nanogratings at a specific location. The pulse energy was adjusted to 300 nJ during the inscription, which is slightly higher than the threshold for the formation of nanogratings and prevents inducing excessive transmission loss. The ultrafast laser enhancement of Rayleigh profiles in optical fibers described in this paper is a general approach, which have been successfully applied to four types of optical fiber with similar Rayleigh enhancement effects and temperature stabilities. These fibers include standard telecommunication fibers (Corning SMF-28), pure silica core fibers (Corning Vascade) and D-shaped fibers with a standard telecommunication fiber core (Corning SMF-28).

## References

[1] Yokokawa H, Tu H, Iwanschitz B, Mai A (2008). Fundamental mechanisms limiting solid oxide fuel cell durability. Journal of Power Sources.

[2] Dikwal CM, Bujalski W, Kendall K (2009). The effect of temperature gradients on thermal cycling and isothermal ageing of micro-tubular solid oxide fuel cells. Journal of Power Sources.

[3] Razbani O, Wærnhus I, Assadi M (2013). Experimental investigation of temperature distribution over a planar solid oxide fuel cell. Applied energy.

[4] Aydın Ö, Nakajima H, Kitahara T (2016). Reliability of the numerical SOFC models for estimating the spatial current and temperature variations. International Journal of Hydrogen Energy.

[5] Bedogni S, Campanari S, Iora P, Montelatici L, Silva P (2007). Experimental analysis and modeling for a circular-planar type IT-SOFC. Journal of Power Sources.

[6] Loranger S, Gagné M, Lambin-Iezzi V, Kashyap R (2015). Rayleigh scatter based order of magnitude increase in distributed temperature and strain sensing by simple UV exposure of optical fibre. Scientific Reports.

[7] Jin J, Zhang H, Liu J, Li Y (2016). Distributed Temperature Sensing Based on Rayleigh Scattering in Irradiated Optical Fiber. IEEE Sensors Journal.

[8] Kreger, S. T., Sang, A. K., Gifford, D. K. & Froggatt, M. E. Distributed strain and temperature sensing in plastic optical fiber using Rayleigh scatter. *Proceedings of SPIE - The International Society for Optical Engineering* **7316**, 73160A-73160A-73168 (2009).

[9] Buric, M., Ohodnicki, P., Yan, A., Huang, S. & Chen, K. P. In *SPIE Optical Engineering + Applications*. 997708.

[10] Smelser CW, Mihailov SJ, Grobnic D (2005). Formation of Type I-IR and Type II-IR gratings with an ultrafast IR laser and a phase mask. Opt. Express.

[11] Xu Y (2015). Optical fiber random grating-based multiparameter sensor. Opt. Lett..

[12] Hnatovsky C (2005). Pulse duration dependence of femtosecond-laser-fabricated nanogratings in fused silica. Applied Physics Letters.

[13] Bricchi E, Kazansky PG (2006). Extraordinary stability of anisotropic femtosecond direct-written structures embedded in silica glass. Applied physics letters.

[14] Richter S (2012). Nanogratings in fused silica: Formation, control, and applications. Journal of Laser Applications.

[15] Zhang J, Gecevičius M, Beresna M, Kazansky PG (2014). Seemingly Unlimited Lifetime Data Storage in Nanostructured Glass. Physical Review Letters.

[16] Shimotsuma Y, Kazansky PG, Qiu J, Hirao K (2003). Self-Organized Nanogratings in Glass Irradiated by Ultrashort Light Pulses. Physical Review Letters.

[17] Taylor R, Hnatovsky C, Simova E (2008). Applications of femtosecond laser induced self‐organized planar nanocracks inside fused silica glass. Laser & Photonics Reviews.

[18] Beresna M, Gecevičius M, Kazansky PG, Taylor T, Kavokin AV (2012). Exciton mediated self-organization in glass driven by ultrashort light pulses. Applied Physics Letters.

[19] Liao Y (2015). High-fidelity visualization of formation of volume nanogratings in porous glass by femtosecond laser irradiation. Optica.

[20] Liao Y (2015). Formation of in-volume nanogratings with sub-100-nm periods in glass by femtosecond laser irradiation. Opt. Lett..

[21] Liang F, Vallée R, Chin SL (2012). Mechanism of nanograting formation on the surface of fused silica. Opt. Express.

[22] Hnatovsky C, Grobnic D, Coulas D, Barnes M, Mihailov SJ (2017). Self-organized nanostructure formation during femtosecond-laser inscription of fiber Bragg gratings. Opt. Lett..

[23] Dai Y, Patel A, Song J, Beresna M, Kazansky PG (2016). Void-nanograting transition by ultrashort laser pulse irradiation in silica glass. Opt. Express.

[24] Rudenko A, Colombier J-P, Itina TE (2016). From random inhomogeneities to periodic nanostructures induced in bulk silica by ultrashort laser. Physical Review B.

[25] Hnatovsky C (2006). Fabrication of microchannels in glass using focused femtosecond laser radiation and selective chemical etching. Appl Phys A.

[26] Kazansky PG (1999). Anomalous Anisotropic Light Scattering in Ge-Doped Silica Glass. Physical Review Letters.

[27] Beresna M, Kazansky PG (2010). Polarization diffraction grating produced by femtosecond laser nanostructuring in glass. Opt. Lett..

[28] Froggatt ME, Gifford DK, Kreger S, Wolfe M, Soller BJ (2006). Characterization of Polarization-Maintaining Fiber Using High-Sensitivity Optical-Frequency-Domain Reflectometry. Journal of Lightwave Technology.

[29] Lu P, Grobnic D, Mihailov SJ (2007). Characterization of the birefringence in fiber Bragg gratings fabricated with an ultrafast-infrared laser. Journal of lightwave technology.

[30] Boffa V, Blank DHA, ten Elshof JE (2008). Hydrothermal stability of microporous silica and niobia–silica membranes. Journal of Membrane Science.

[31] Froggatt M, Moore J (1998). High-spatial-resolution distributed strain measurement in optical fiber with Rayleigh scatter. Appl. Opt..

[32] Wang T, Shao L-Y, Canning J, Cook K (2013). Temperature and strain characterization of regenerated gratings. Opt. Lett..

[33] Soller BJ, Gifford DK, Wolfe MS, Froggatt ME (2005). High resolution optical frequency domain reflectometry for characterization of components and assemblies. Opt. Express.

